# Hyperthermia Potentiates the Effectiveness of Anticancer Drugs—Cisplatin and Tamoxifen on Ovarian Cancer Cells In Vitro

**DOI:** 10.3390/ijms252413664

**Published:** 2024-12-20

**Authors:** Aleksandra Zoń, Ilona Anna Bednarek

**Affiliations:** Department of Biotechnology and Genetic Engineering, Faculty of Pharmaceutical Sciences in Sosnowiec, Medical University of Silesia, Jedności 8, 41-200 Sosnowiec, Poland

**Keywords:** ovarian cancer, hyperthermia, combination therapy, cisplatin, tamoxifen

## Abstract

Ovarian cancer is one of the most prevalent cancers among women. Due to the frequent problems during treatment, such as relapses or the development of resistance to treatment, new methods of treating this disease are being sought. A special attention is directed towards the combination therapies combining several different anticancer agents. The aim of the following study was to examine the effect of combination therapy with mild hyperthermia (temperatures of 39 °C and 40 °C) and anticancer drugs—cisplatin and tamoxifen—on the SKOV-3 ovarian cancer cell line in vitro. Furthermore, the study also assessed the effect of moderate hyperthermia on the anticancer effectiveness of both of these drugs. The cytotoxic effect of the therapy was assessed using MTT assay and fluorescent acridine orange staining. Changes in the expression of genes involved in apoptosis processes were evaluated using RT-qPCR. It has been shown that the use of combination therapy leads to a significant increase in apoptosis processes in SKOV-3 ovarian cancer cells and, consequently, to a decrease in their viability. At the molecular level, mild hyperthermia leads primarily to a decrease in the expression of anti-apoptotic genes, and also, to a small extent, to an increase in the expression of proapoptotic genes. The results also indicate that moderate hyperthermia has a positive effect on the cytotoxic efficacy of both cisplatin and tamoxifen on ovarian cancer cells. This suggests that hyperthermia could be a potential component in combination therapy for ovarian cancer.

## 1. Introduction

Ovarian cancer is the eighth most-common cancer in women worldwide [1]. It is estimated that every year this disease is diagnosed in over 320,000 women, and over 206,000 women die of it, which constitutes almost 4.8% of all cancer deaths in the world among women [1]. This type of cancer predominantly affects women aged 55–64 (24.5% of all cases), although there is an increasing prevalence among younger women—over 45 years of age (16% of cases). The highest mortality rate is observed among women aged 65–74 (29.1% of deaths) [2].

The lack of specific screening tests and the asymptomatic course of the disease in its early stages result in approximately 70% of ovarian cancer cases being detected in an advanced stage (FIGO (The International Federation of Gynaecology and Obstetrics) stage III–IV) [3,4,5]. This fact results in a significant reduction in chances of a full recovery—the five-year survival rate in the case of detection of the disease in stage I is 70–80%, while in stage IV, it is drastically reduced to only 15% [4,5].

The standard treatment for advanced ovarian cancer includes a surgical removal of the tumor mass, followed by a chemotherapy based on platinum and taxane compounds [6,7]. One of the most common drugs used in the treatment of ovarian cancer is cisplatin. The mechanism of its cytotoxic action is based primarily on the activation of apoptosis processes in cancer cells, mainly through binding to the genomic and mitochondrial DNA of these cells—thus leading to their damage, as well as inducing the formation of oxygen free radicals in these cells [8].

An additional important factor for the process of carcinogenesis in relation to ovarian cancer is its relationship with hormonal regulation. According to research, steroid hormones produced by the hypothalamic–pituitary–ovarian axis probably play an important role in the development of this cancer [9]. Particular attention is paid to estrogens, which, by acting through estrogen receptors presented on ovarian cancer cells, participate in the regulation of the proliferation and differentiation of these cells, positively influencing the cancer progression [10]. Therefore, the attempts of using modulators of hormone-dependent receptor signal transduction, e.g., tamoxifen, in the therapy of ovarian cancer, seem to be important. Tamoxifen is a non-steroidal estrogen receptor antagonist that, by blocking the estrogen binding site on the receptor, inhibits the pro-tumor effects of estrogens [11]. Tamoxifen also has the ability to induce apoptosis in cancer cells, mainly by increasing the expression level of pro-apoptotic genes encoding proteins that mediate the activation of signaling pathways such as Tp53or MAPK [11].

Unfortunately, standard methods of ovarian cancer treatment rarely lead to a complete cure of the disease—its recurrence is observed in 25% of patients with disease detected in stage I, and in as many as 80% of patients with the disease detected in more advanced stages [12].

Due to the relatively low effectiveness of standard methods of ovarian cancer therapy, as well as the frequent development of resistance to the used drugs, new methods of treating this disease are being intensively sought [6]. One of these methods is a combination therapy using cytotoxic drugs or radiotherapy, combined with hyperthermia—the use of a temperature several degrees higher than the physiological body temperature, up to 39–45 °C [13,14].

The molecular effects induced by elevated temperature in treated cells, which enable its use in combination with chemotherapeutics, include, for example, increased intracellular drug transport and accumulation, as well as the limitation of detoxification potential in cancer cells, e.g., by reducing the level of antioxidants, especially glutathione [13,15,16,17]. Moreover, it has also been shown that increased temperature inhibits the repair processes of DNA damages caused by the cytotoxic drugs [18,19,20]. Under hyperthermic conditions, the degradation process of proteins responsible for DNA repair in homologous recombination (primarily the BRCA2 protein) is intensified, which leads to the inhibition of DNA repair systems [19,20].

At this point, it is also important to consider the fact that cells exposed to hyperthermia can activate self-protective processes, such as autophagy. The aggregates of dysfunctional proteins formed under the influence of a thermal stimulus are removed from cells based on autophagy mechanisms, which can ultimately protect cells from death in response to the applied hyperthermia [21].

The aim of the following study was to investigate the impact of mild hyperthermia on the viability and apoptosis processes in ovarian cancer cells in vitro, as well as to assess whether the combined use of cisplatin and hyperthermia enhances the efficacy of this drug. This study also included the effects of the estrogen receptor inhibitor tamoxifen as a modulator of the cell response to the tested agents.

Because of the possibility of autophagy process activation by the studied factors, the induction of this process in SKOV-3 cells under the influence of tested drugs, as well as the effect of moderate hyperthermia on the intensity of this phenomenon, were also assessed. Taking into account the potential mechanisms of action of the used factors, we have also verified the expression level of genes associated with apoptosis processes.

## 2. Results

### 2.1. Assessment of the Cytotoxic Effects of Drugs and Mild Hyperthermia on SKOV-3 Cells (MTT Assay)

**Figure 1:**
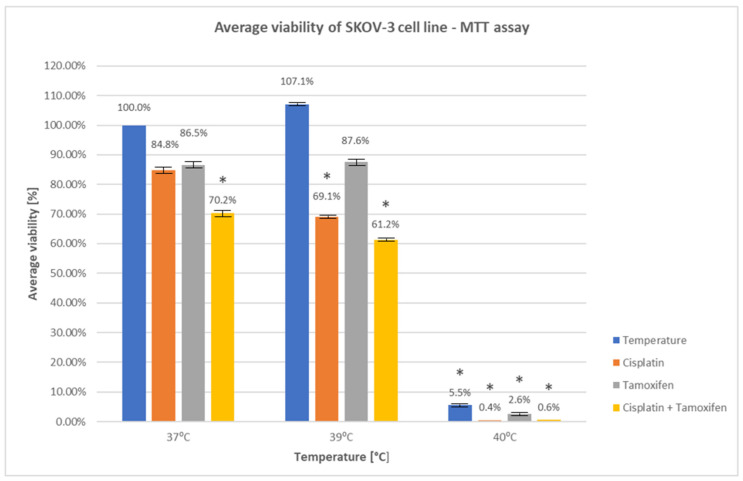
Average percentage viability of SKOV-3 cells treated with mild hyperthermia for 24 h and drugs—MTT assay. The statistically significant (*p* < 0.05) differences in the average percentage of cell viability were marked with an asterisk.

In order to assess the influence of drugs, hyperthermia, and both of these factors together on the viability of SKOV-3 line cells, the MTT assay was performed. The control group consisted of cells cultured at 37 °C without the addition of drugs—their viability was assumed to be 100%. The MTT assay results are presented in Figure 1 and Table S1 (Supplementary Materials).

The obtained results show that treating SKOV-3 cells with tested drugs in a standard temperature of 37 °C led to a decrease in their viability. The biggest decrease in viability, in comparison to the control (1.06 ± 0.03), was observed for cells treated with cisplatin and tamoxifen (0.74 ± 0.08) (*p* < 0.05). A treatment with cisplatin (0.90 ± 0.11) or tamoxifen (0.91 ± 0.22) only also led to a decrease in cell viability, but not to such a degree as a treatment with both of these drugs in combination (the differences did not show a statistical significance; the differences were *p* = 0.15 for cells treated with cisplatin and *p* = 0.24 for cells treated with tamoxifen).

For cells treated only with an elevated temperature of 39 °C for 24 h, a slight increase in viability was observed (1.13 ± 0.04). The increase was not statistically significant (*p* = 0.91).

Moreover, it has also been shown that the simultaneous treatment of SKOV-3 cells with a temperature of 39 °C for 24 h and drugs leads to a significant decrease in their viability. The effect was particularly visible for cisplatin (0.73 ± 0.05) (*p* < 0.05) and for cisplatin in combination with tamoxifen (0.65 ± 0.03) (*p* < 0.05). This effect was not that clear when just using tamoxifen (0.93 ± 0.08) (*p* = 0.32).

However, the biggest reduction in cell viability was observed for treatment with a temperature of 40 °C. An exposure of cells to 40 °C for 24 h led to a drastic decrease in their viability (0.06 ± 0.004) in comparison to the control (*p* < 0.05), whereas combinatory treatment with temperature and drugs gave the following results: cisplatin (0.004 ± 0.01), tamoxifen (0.03 ± 0.02), and both of these drugs (0.006 ± 0.01), leading to almost complete cell death (*p* < 0.05 in all cases).

The best combined cytotoxic effect of drugs and temperature was observed for the combination of cisplatin + tamoxifen + temperature (both 39 °C and 40 °C); the weakest effect was from the combination of tamoxifen + temperature.

The obtained results indicate that treating ovarian cancer cells with combination therapy based on drugs with an addition of mild hyperthermia leads to a significant increase in their cytotoxic properties.

### 2.2. Assessment of Morphological Changes in SKOV-3 Cells Using Fluorescent Staining

**Figure 2:**
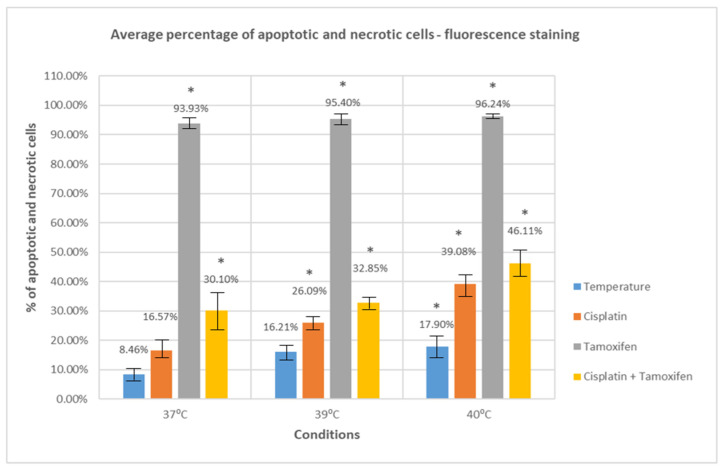
Average percentage of apoptotic and necrotic cells—SKOV-3 cells treated with moderate hyperthermia and drugs. The statistically significant (*p* < 0.05) differences in the average percentage of apoptotic and necrotic cells were marked with an asterisk.

To further investigate the effect of selected drugs as well as thermal factor on SKOV-3 line ovarian cancer cells, the assessment of changes in their morphological features using the fluorescence staining technique with acridine orange was performed. Acridine orange is a fluorescent dye that penetrates the cell membrane and binds, through intercalation, to the DNA and RNA of cells. Based on specific morphological features, typical for normal, apoptotic, and necrotic cells, the average percentage of apoptotic and necrotic cells was determined for the control and each of the tested samples. The results are presented in Figure 2 and Table S2 (Supplementary Materials).

**Figure 3:**
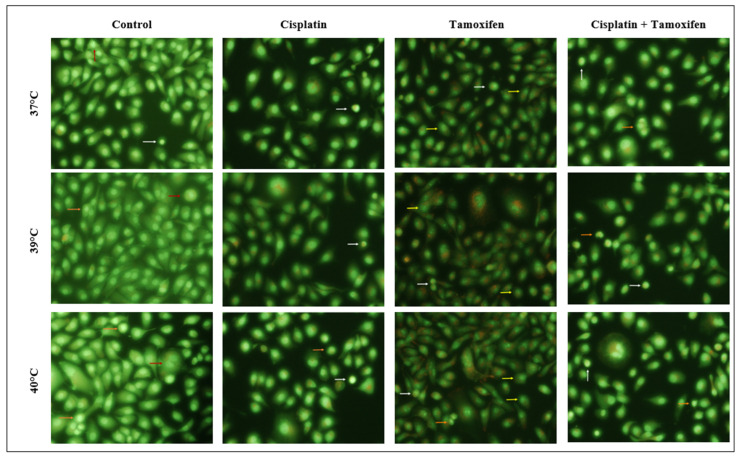
Cells treated with mild hyperthermia and drugs, stained with acridine orange. Arrows indicate cells with characteristic features of apoptosis and necrosis processes.

The morphological features that were considered as typical for normal, healthy cells were as follows: round, regular cell shape, a round nucleus located in the central part of the cell that emits moderately intense green fluorescence, and the presence of an intact cell membrane. Characteristic features indicating the processes of early apoptosis in cells were as follows: a significant reduction in cell volume, the condensation of the cell nucleus, and its very strong green-yellow fluorescence. Characteristic features of late apoptosis included even stronger condensation and the fragmentation of the cell nucleus (emitting strong green fluorescence), as well as the disruption of cell membranes and formation of apoptotic bodies. Cells classified as necrotic were characterized by a significant increase in their volume as well as the fragmentation of their cell membrane and cell nucleus. The examples of cells classified into particular groups are presented in the photos in Figure 3. White arrows indicate cells showing features characteristic for the early apoptosis processes, the orange arrows—cells with characteristics of late apoptosis, and the red arrows—cells with characteristic necrotic features (the examples shown in the group of control cells). Additionally, the yellow arrows indicate tamoxifen-treated cells showing features characteristic of apoptosis, with an additional strong fragmentation of the nucleus.

Our results show that treating SKOV-3 cells with tested drugs at a temperature of 37 °C leads to an increase in the number of apoptotic and necrotic cells compared to the control (untreated cells, cultured at 37 °C) (8.46 ± 2.46). The increase observed for cisplatin was minimal, approximately 8%, and did not reach statistical significance (16.57 ± 3.51) (*p* = 0.08). A significant increase in the number of apoptotic and necrotic cells was observed for tamoxifen (93.93 ± 1.64) (*p* < 0.05). Importantly, cells in which the apoptosis process was induced with tamoxifen did not show the same characteristic features as cells treated with cisplatin or hyperthermia. Instead, they were characterized by spectacular changes in morphology, manifested by a very strong fragmentation of the cell nucleus. The examples of tamoxifen-treated cells showing those features are presented in Figure 3.

Additionally, the treatment of SKOV-3 cells with both cisplatin and tamoxifen at 37 °C (30.10 ± 7.59) also led to increased apoptosis and necrosis processes compared to the control (*p* < 0.05).

Treating cells with an elevated temperature of 39 °C resulted in a slight increase in the number of apoptotic and necrotic cells in comparison to the control cells (16.21 ± 3.30), but the increase was not statistically significant (*p* = 0.11). A relevant increase in a number of cells presenting apoptotic and necrotic features was observed for simultaneous treatment with tested drugs and a temperature of 39 °C (*p* < 0.05 for both tested drugs and their combination). The increase was most noticeable for cells treated with tamoxifen (95.40 ± 1.630) as well as for cells treated with cisplatin and tamoxifen (32.85 ± 2.54).

The most significant change in the number of apoptotic and necrotic cells was observed for cells treated with elevated temperature of 40 °C, both in the case of treatment only with temperature (17.90 ± 4.03) as well as with temperature combined with cisplatin (39.08 ± 4.06), tamoxifen (96.24 ± 0.85), and with both cisplatin and tamoxifen (46.11 ± 4.36). All these changes were statistically significant (*p* < 0.05).

The biggest difference in the number of apoptotic and necrotic cells in comparison to control was observed for cells treated with tamoxifen and a temperature of 40 °C—the increase was over 87% (*p* < 0.05).

The greatest impact of temperature on the effectiveness of the drug’s cytotoxicity was observed for cisplatin; the difference in the number of apoptotic and necrotic cells between cells treated with cisplatin in 37 °C and 39 °C was 22.51% and was statistically significant (*p* < 0.05). A significant difference in effectiveness was also observed for the combination of cisplatin and tamoxifen at 40 °C, with a difference of 16.01% in the number of apoptotic and necrotic cells between this temperature and 37 °C.

The results obtained in this part of the experiment also indicate that the simultaneous use of cytotoxic drugs with mild hyperthermia in the therapy of ovarian cancer leads to a significant increase in their anticancer activity.

### 2.3. Assessment of the Induction of Autophagy Processes in SKOV-3 Cells Treated with the Tested Factors Using Acridine Orange as a Ratio Metric Biosensor of Autophagosomes

Acridine orange is a basic fluorescent dye that, upon penetration into acidic cellular organelles, undergoes protonation and dimerization, resulting in the organelles’ orange/red coloration. The well-known low-pH organelles are autophagolysosomes—acidic vesicular organelles (AVOs)—that are formed in cells during the autophagy process. An increase in the number of autophagolysosomes in cells that is observed during the intensification of autophagy processes leads to an increase in the emission of red fluorescence by cells stained with acridine orange. The determination of red fluorescence intensity to green fluorescence intensity ratio (R/GFIR) allows for the assessment of autophagy processes intensity in the studied cells.

**Figure 4:**
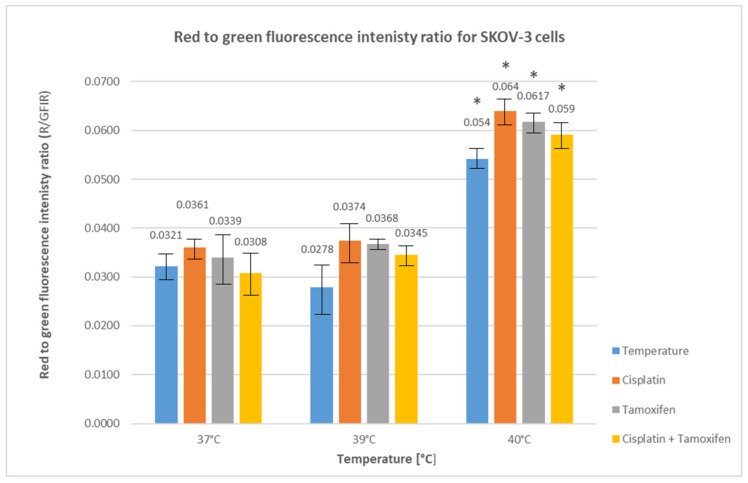
Average red fluorescence to green fluorescence intensity ratio for SKOV-3 cells treated with moderate hyperthermia and drugs. Statistically significant differences (*p* < 0.05) in the average ratio are marked with an asterisk.

In order to more precisely determine the type of death occurring in cancer cells treated with hyperthermia and drugs, the cells were stained with acridine orange, and the red and green fluorescence emitted by the cells was measured. Then, the ratio of red to green fluorescence intensity (R/GFIR) was calculated. The obtained results are presented in Figure 4.

It was shown that the use of cisplatin and tamoxifen at the standard temperature of 37 °C in SKOV-3 cells treatment led to a slight increase in the average red fluorescence to green fluorescence intensity ratio (0.036 ± 0.002 for cisplatin and 0.0339 ± 0.006 for tamoxifen) compared to the control cells (0.0321 ± 0.003). However, these differences were not statistically significant (*p* = 0.76 for cisplatin and *p* = 0.99 for tamoxifen). The simultaneous use of both drugs at this temperature led to a slight decrease in this parameter compared to the control (0.0308 ± 0.005, *p* = 0.99).

The exposure of SKOV-3 cells to the higher temperature of 39 °C led to a slight decrease in R/GFIR value (0.0278 ± 0.006), compared to the control, but the increase was not statistically significant (*p* = 0.76). The use of the tested drugs at this temperature, both in monotherapy and in combination, led to a slight increase in the R/GFIR value compared to the control (R/GFIR = 0.0374 ± 0.004 for cisplatin; 0.0368 ± 0.001 for tamoxifen; 0.0345 ± 0.002 for both drugs used simultaneously, *p* > 0.05 in all cases).

A significant increase in the red fluorescence to green fluorescence intensity ratio was observed when cells were treated with a temperature of 40 °C—both with only a temperature (0.054 ± 0.002) and with temperature together with drugs. The highest increase in R/GFIR values was observed for cells treated with cisplatin (0.064 ± 0.003), slightly lower for cells treated with tamoxifen (0.0617 ± 0.002) and with both drugs simultaneously (0.059 ± 0.003). The increase in all R/GFIR values was statistically significant (*p* < 0.05).

The biggest increase in the average ratio of red fluorescence to green fluorescence intensity was observed (at each temperature point) for cells treated with cisplatin, which suggests that it has the strongest ability of the studied drugs to induce autophagy processes in the studied cells. At the same time, it was also shown that elevated temperatures, especially 40 °C, have a positive effect on the ability to induce autophagy processes with the studied drugs used both independently and in combination.

### 2.4. Assessment of Changes in the Expression of Apoptotic-Related Genes in SKOV-3 Cells Using RT-qPCR

To analyze changes in the expression of genes involved in apoptosis processes occurring in SKOV-3 cells treated with drugs and mild hyperthermia, RT-qPCR, using an RT2 Profiler PCR Array Human Apoptosis kit, was performed. We have analyzed genes belonging to the pro- and anti-apoptotic groups, as well as genes regulating apoptosis processes, and genes encoding programmed cell death receptors and cell death executors—caspases.

**Figure 5:**
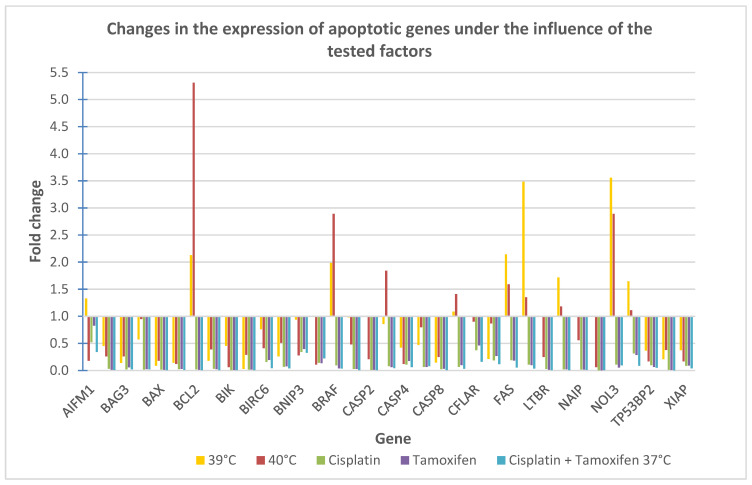
Changes in the expression of selected genes involved in apoptosis processes, induced by the tested factors.

**Figure 6:**
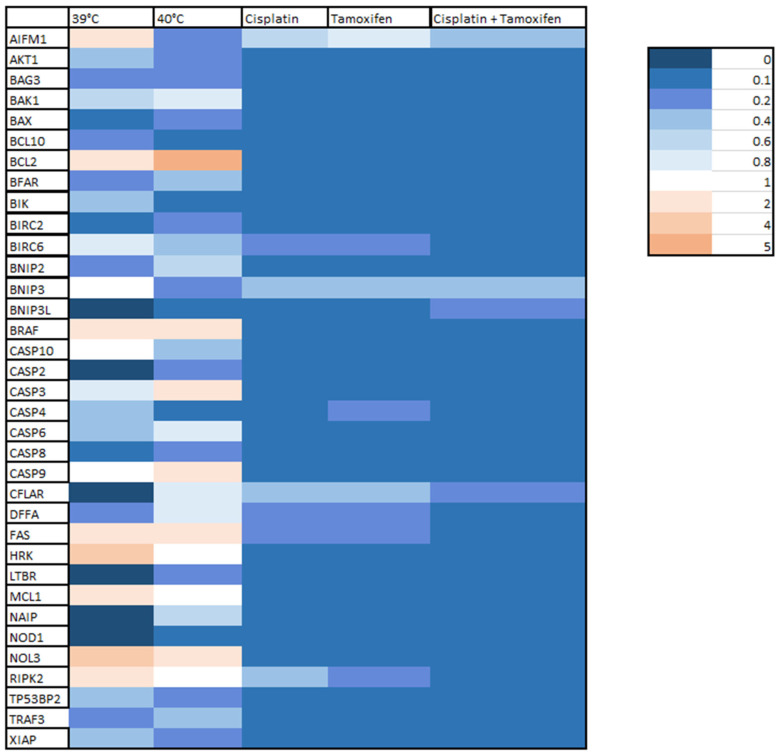
Heat map showing changes in the expression of selected genes involved in apoptosis under the influence of the tested drugs and hyperthermia.

The results of the analysis are presented in Figure 5 as fold changes in selected genes. Fold change values above 1 indicate an up-regulation of gene, while fold change values below 1 indicate a gene down-regulation. Additionally, the obtained results are presented in Figure 6 in a form of a heat map.

The analysis of changes in the expression of genes involved in apoptosis processes revealed a significant down-regulation of genes belonging to the group of apoptosis inhibitors, such as AKT1, BAG3, BCL10, BFAR, BIRC2, BIRC6, BNIP2, and XIAP, both in cells treated with mild hyperthermia (39 °C and 40 °C), as well as those treated with the tested drugs. The most significant decrease in expression in this group of genes was observed for cells treated with cisplatin for the BAG3 gene or with cisplatin in combination with tamoxifen for all other mentioned genes.

Moreover, for cells treated with a temperature of 40 °C, as well as cells treated with drugs, a decrease in expression of another two anti-apoptotic genes—CFLAR and NAIB—was observed. Also, for these genes, the biggest decrease in expression was observed for cells treated with cisplatin and tamoxifen.

Additionally, a significant down-regulation of other genes belonging to the family of apoptosis inhibitors, BCL2, BRAF, MCL1 and NOL3, was observed in cells treated with cisplatin, tamoxifen, and their combination. The biggest decrease in their expression was also noted for cells treated with cisplatin and tamoxifen in combination. However, in cells treated only with hyperthermia (39 °C and 40 °C), the opposite effect was observed—the expression of the above-mentioned genes was up-regulated.

What is more, for cells treated with temperature of 39 °C and 40 °C, an up-regulation of a few genes involved in the induction of apoptosis processes, CASP9, FAS, HRK, RIPK2, was observed. An increase in expression was also observed for other proapoptotic genes: AIFM1—in cells treated with temperature of 39 °C, and CASP3—in cells treated with 40 °C. In cells treated with drugs, the genes expression of those genes was down-regulated.

For other genes belonging to the “proapoptotic” group such as caspases genes: CASP2, CASP4, CASP6, CASP8, CASP10; Bcl-2 family genes: BAK1, BAX, as well as BIR, BNIP3, BNIP3L, and DFFA, LTBR, NOD1, TP53BP2 or TRAF3 genes, despite hyperthermia or drug treatment, a decrease in their expression was observed.

As shown in Figure 4, for cells treated with mild hyperthermia of 39 °C, an increase in the expression of the AIFM1, BCL2, BRAF, FAS, HRK, MCL1, NOL3, and RIPK2 genes was observed. The most significant increase in expression was noted for the NOL3 gene. The expression of other tested genes remained unchanged (BNIP3, CASP10, CASP9) or was significantly decreased. The biggest down-regulation was observed for the BIRC2 gene. The complete lack of expression was demonstrated for the following genes: BNIP3L, CASP2, CFLAR, LTBR, NAIP and NOD1.

For cells treated with hyperthermia at 40 °C, a strong up-regulation was observed for the BCL2 gene, as well as for the BRAF, CASP3, CASP9, FAS, and NOL3 genes. No differences in expression were demonstrated for the HRK, MCL1, and RIPK2 genes. For the remaining genes, a decrease in expression level was noted.

For cells treated with cisplatin, tamoxifen, and a combination of those drugs, no increase in expression of any of the studied genes was observed—all of the genes were down-regulated. The strongest decrease in expression was observed for the NOD1 gene for cells treated with cisplatin and cells treated with tamoxifen, and for the MCL1 and TRAF3 genes, for cells treated with both of these drugs combined.

## 3. Materials and Methods

### 3.1. Cell Culture

For our research, the ovarian cancer eukaryotic cell line SKOV-3, purchased from the American Type Culture Collection (ATCC:HTB-77™), was used. This line was obtained by isolating ovarian cells from a 64-year-old Caucasian woman suffering from ovarian adenocarcinoma. SKOV-3 line is considered a cisplatin-resistant line.

SKOV-3 line cells were cultured in DMEM+ GlutaMAX™ medium (Gibco, Thermo Fisher Scientific, Waltham, MA, USA) supplemented with 10% fetal bovine serum (PAN Biotech, Aidenbachch, Germany) and gentamycin (final concentration 50 μg/mL of medium; Sigma Aldrich, St. Louis, MO, USA) under the following conditions: temperature 37 °C, humidity 95%, 5% CO_2_.

### 3.2. Assessment of the Mild Hyperthermia’s Potential of Thermal Chemo Sensitization of SKOV-3 Cells to Tested Drugs 

SKOV-3 cells were exposed to the following selected agents: mild hyperthermia (39 °C and 40 °C) and drugs—cisplatin and tamoxifen. The effects of the used modulators were verified in terms of cytotoxic effect (MTT assay) and in terms of morphological changes indicating apoptosis processes in cells (acridine orange staining). Additionally, the possible induction of autophagy processes under the influence of the tested factors was also assessed by determining the ratio of red to green fluorescence intensity in cells stained with acridine orange.

#### 3.2.1. Assessment of Apoptosis Processes Induced by Drugs and Moderate Hyperthermia in SKOV-3 Cells

The cytotoxic activity of the selected drugs and temperature was tested with the MTT assay—a colorimetric assay that is based on the reduction in yellow tetrazolium salts (MTT) to water-insoluble violet formazan, by viable, metabolically active cells. The amount of the resulting product is proportional to the number of viable cells.

In order to stimulate cells to apoptosis, SKOV-3 line cells were seeded in three 96-well plates (seeding density: 1 × 10^4^ cell/well; SARSTEDT, Nümbrecht, Germany) and incubated for 24 h in standard environmental conditions. To stimulate the apoptosis processes, the following drugs were used: cisplatin (cis-Dichlorodiammine platinum(II); Sigma Aldrich, St. Louis, MO, USA) at a stock concentration of 10 μg/μL, tamoxifen ((Z)-1-(p-Dimethylaminoethoxyphenyl)-1,2-diphenyl-1-butene; Sigma Aldrich, St. Louis, MO, USA) at a stock concentration of 0.1 μg/μL, and a mixture of both of these drugs at the same stock concentrations. The medium for apoptosis stimulation was prepared by mixing the drug’s stock solutions with 10% DMEM medium in such a ratio so that the drug’s final concentration was 50 nM/mL for cisplatin and 1 nM/mL for tamoxifen (both for drugs used separately and in combination). To test the cytotoxic effect of hyperthermia used independently, tested cells were in incubated in pure DMEM medium (without any drugs). Each sample was prepared in triplicate.

The plates were incubated at 3 different temperature points: 37 °C, 39 °C, and 40 °C, for 1 h and 24 h.

The stock solution of the 3-(4,5-Dimethyl-2-thiazolyl)-2,5-diphenyl-2H-tetrazolium bromide (MTT; Sigma Aldrich, St. Louis, MO, USA) at a concentration of 5 mg/mL was mixed with DMEM medium without phenol red (Gibco, Thermo Fisher Scientific, Waltham, MA, USA) in the following proportion: 500 μL of MTT stock solution per 10 mL of medium. The resulting staining solution was added to the cells and incubated at 37 °C for 2 h. After incubation, the MTT solution was removed and 99.9% DMSO (Sigma Aldrich, St. Louis, MO, USA) was added to dissolve the formazan crystals. After incubation in 37 °C for 15 min, the absorbance was read at λ = 570 nm and λ = 630 nm (reference wavelength) wavelengths using a Biotek EPOCH plate reader (Agilent Technologies, Santa Clara, CA, USA).

#### 3.2.2. Assessment of Morphological Changes Specific for Apoptosis Processes in SKOV-3 Cells Using Fluorescence Staining

To assess the morphological changes in SKOV-3 cells after treatment with the selected drugs and mild hyperthermia, the cells were seeded in three 6-well plates (seeding density: 2 × 10^5^ cell/well; SARSTEDT, Nümbrecht, Germany) and incubated for 24 h in standard conditions. After incubation, cells were synchronized by serum starvation for 24 h. Then, to stimulate the apoptosis processes, cells were treated with the same drugs in the same final concentrations as described in Section 3.2.1. Also, in this case, to assess the cytotoxic effect of hyperthermia alone, some of the cells were incubated in pure DMEM medium (without the addition of drugs).

Pre-incubation in elevated temperatures (39 °C and 40 °C) was carried out for 1 h and then the cells were transferred to 37 °C for 24 h. The control cells were incubated only at 37 °C for 24 h. The next day, the cells were washed with DMEM medium without phenol red, stained with acridine orange (Thermo Fisher Scientific, Waltham, MA, USA) at a final concentration of 10 μg/mL, and incubated for 2–3 min at room temperature. After incubation, the staining solution was removed and the cells were washed again with DMEM medium without phenol red. Prepared cells were observed using an Eclipse Ti fluorescence microscope (filters with parameters Ex/Em = 500/526; Nikon Instruments Inc., Melville, NY, USA). The photos were taken using 10× and 20× lenses.

### 3.3. Assessment of the Induction of Autophagy Processes in SKOV-3 Cells Treated with the Tested Factors Using Acridine Orange as a Ratio Metric Biosensor of Autophagosomes

To assess the autophagy processes in SKOV-3 cells treated with tested drugs and mild hyperthermia, cells were seeded in three 96-well plates (seeding density: 1 × 10^4^; SARSTEDT, Nümbrecht, Germany) and cultured to reach 80% confluence. Then, the cells were synchronized by serum starvation for 24 h. After synchronization, cells were treated with drugs and moderate hyperthermia in the same manner as described in Section 3.2.2. The next day, cells were washed twice with PBS (Gibco, Thermo Fisher Scientific, Waltham, MA, USA) and stained with acridine orange (final concentration 2.5 μg/mL) for 15 min at 37 °C. After the incubation with a dye, cells were washed twice with PBS, and then the emission of green (Ex/Em = 485/535) and red (Ex/Em = 465/650) fluorescence was measured using TECAN Spark 10M reader (Tecan Group Ltd., Männedorf, Switzerland).

### 3.4. Assessment of Changes in Gene Expression Induced by Drugs and Moderate Hyperthermia in SKOV-3 Cells (RT-qPCR)

To assess changes in the expression level of genes associated with apoptosis processes in SKOV-3 cells treated with tested agents, RT-qPCR, using an RT2 Profiler PCR Array Human Apoptosis kit (QIAGEN N.V., Hilden, Germany), was carried out. A number of genes belonging to the different groups were of the following types: genes involved in the induction, inhibition, and regulation of apoptosis processes, genes encoding caspases and their regulators, or genes encoding programmed death receptors.

SKOV-3 line cells were seeded in cell culture flasks (SARSTEDT, Nümbrecht, Germany) and cultured in typical environmental conditions (as described in Section 2.2) until 85% confluence. Then, some of the cells were incubated for 1 h at three different temperature points: 37 °C (control), 39 °C, and 40 °C—without the addition of drugs—and the others were treated with cisplatin, tamoxifen, and a combination of both of these drugs, at final concentrations identical to those described in Section 3.2.1. All cells were incubated for another 24 h at 37 °C.

Then, 24 h after stimulation, total RNA was isolated using the ZR-Duet DNA/RNA MiniPrep kit (ZYMO RESAEARCH, Irvine, CA, USA) according to the manufacturer’s instructions. The quality and purity of the isolated RNA were checked spectrophotometrically using an BioPhotometer spectrophotometer (Eppendorf, Hamburg, Germany) by determining the A260/280 ratio (obtained values in the range of 2.0–2.2 for all samples) and via agarose electrophoresis.

The RNA was reverse transcribed to cDNA using the RT2 First Strand Kit (QIAGEN N.V., Hilden, Germany) according to the manufacturer’s instructions. A mix of the cDNA with RT2 SYBR Green ROXTM qPCR Mastermix (QIAGEN N.V., Hilden, Germany) was prepared according to the manufacturer’s protocol and portioned out into the wells of the RT2 Profiler PCR Array Human Apoptosis plate. The PCR reaction was performed using the Mx3000p system (Stratagene, San Diego, CA, USA) according to the manufacturer’s recommended thermal profile: the activation of HotStart DNA Taq Polymerase for 10 min at 95 °C, followed by 40 cycles of 15 s at 95 °C, 1 min at 60 °C, and then 10 min at 72 °C. The average expression of selected genes was calculated using the ΔΔCt method. As the reference gene, the hypoxanthine phosphoribosyltransferase 1 (HPRT1) gene was used.

### 3.5. Statistical Analysis

A statistical analysis of all the results obtained during our research was performed using the Statistica 13.3 Software (TIBCO Software, Palo Alto, CA, USA). To assess the types of distributions, the Shapiro–Wilk test and quantile–quantile plots were used. Quantitative data were presented as mean ± standard deviation. The comparison between experimental groups was based on one-way ANOVA. The homogeneity of variance was assessed using Leven’s test. In order to determine significant differences between the test groups and the control group, post hoc tests were performed (Dunnett’s test). Results were considered as statistically significant if they had a test probability value of *p* < 0.05.

## 4. Discussion

Hyperthermia is a method of oncological treatment that involves the use of heat in cancer therapy. According to the way of delivering heat to cancer tissues, hyperthermia can be divided into three types: local, regional, and systemic hyperthermia [22,23,24]. Hyperthermia is rarely used in a cancer treatment independently—it is usually used as an element of combination therapy with chemotherapy, radiotherapy, or immunotherapy [22,23]. In recent years, there has been growing interest in the use of hyperthermia in oncological disease treatment, which results in an increase in the number of clinical trials using this technique. In 2020, the number of clinical trials using hyperthermia amounted to 235 registered studies [25]. The attempts to use hyperthermia in therapy mainly apply to cervical cancer [25,26,27], breast cancer [28], bladder cancer [25,29], and prostate cancer [30], as well as gastrointestinal cancer [25,31,32]. Great interest is also generated by the use of hyperthermia in the treatment of ovarian cancer—mainly in HIPEC (Hyperthermic Intraperitoneal Chemotherapy) [25]. HIPEC involves the introduction of a cytotoxic drug heated to 40–45 °C (mostly cisplatin, carboplatin, or doxorubicin) into the patient’s peritoneal cavity (immediately after cytosurgical surgery), leaving it for about 90 min and then removing it from the patient’s body [33]. Presumably, the use of elevated temperature in the described therapy increases the penetration of chemotherapeutic agents into cancer cells, intensifies their cytotoxic effect, and reduces the resistance of cancer cells [34].

Opinions on the efficacy of HIPEC in the treatment of ovarian cancer are mixed. A large part of the conducted studies indicates a positive effect of this therapy on the overall survival (OS) and progression-free survival (PFS) of treated patients [35]. One of the most important studies on this topic was the OVHIPEC-1 study conducted by van Driel et al., the results of which indicated an extension of the average 5-year progression-free survival and the 5-year overall survival in women who underwent HIPEC [35,36].

The positive impact of the described therapy on the average 3-year overall survival rate of the women treated with HIPEC was also confirmed in the studies conducted by Lei et al. [35,37].

However, in 2021, a study appeared questioning the effectiveness of HIPEC in the treatment of recurrent ovarian cancer, indicating a reduction in the average overall survival time in the group of women undergoing HIPEC compared to the group undergoing different treatment [38,39].

The lack of complete certainty as to the effectiveness of therapy combining the use of elevated temperature and cytotoxic drugs indicates the need for additional research in this area.

In the following article, we have examined the effect of the therapy combining mild hyperthermia with drugs—cisplatin and tamoxifen—on the SKOV-3 ovarian cancer line. The cytotoxic effect of the therapy was assessed using the MTT assay. In the first stage, we have verified the time of treating SKOV-3 cells with elevated temperature—the assessment was carried out for the period of 1 h and 24 h. Due to the characteristics of the MTT test, which is based on the measurement of metabolic activity of cells, we have decided to choose a longer exposure to elevated temperature so that it would be possible to observe changes in mitochondrial dehydrogenase activity, which occur in cells over time.

It was shown that 24 h treatment of SKOV-3 cells with an elevated temperature of 39 °C led to an increase in cell viability (by approx. 7.1%) compared to the control, and a treatment with a temperature of 40 °C led to a significant decrease in viability by over 94%.

The slight increase in the viability of cells treated with 39 °C may result from the increased production of heat shock proteins (HSP) in these cells after the exposure to an elevated temperature.

Heat shock proteins are a family of proteins that are up-regulated in cellular stress conditions caused by, for example, starvation, hypoxia, or a rapid increase or decrease in temperature. HSPs act as chaperones, preventing the improper folding and aggregation of proteins in cells under stress conditions, thus protecting them from death [40,41].

By interacting with proteins involved in apoptosis pathways, HSPs also play an important role in the course of apoptosis processes in cells. As indicated by numerous studies, there is a significant increase in the expression of these proteins in cancer cells compared to normal cells, which may contribute to the inhibition of apoptosis processes in cancer cells, thus facilitating their survival and the further development of the disease [40,42,43].

Treating cancer cells with an elevated temperature of 39 °C and higher can lead to an increase in the level of heat shock proteins, including HSP27 and HSP70, which exhibit anti-apoptotic properties in those cells [40]. The HSP70 protein acts as a regulator of apoptosis, which, by blocking the release of cytochrome C from mitochondria, inhibits the activation of caspases, thus leading to the inhibition of the apoptosis process [40]. It has been proven that the activation and increase in the level of this protein under cellular stress conditions protects cancer cells from protein damage induced by hyperthermia [44]. Another negative regulator of apoptosis is the HSP27 protein, which has the ability to inhibit apoptosis processes both in the intrinsic pathway (by inhibiting the activity of the Bax protein and by binding to cytochrome C) and the extrinsic pathway (by binding to the DAXX protein) [45,46]. In a study conducted in 2020, Kong et al. showed that treatment of SKOV-3 cells with moderate hyperthermia (39–43 °C) leads to an increased expression of the gene encoding HSP27 protein. This increase translates into increased cell viability and is especially noticeable for cells treated at 39 °C. At higher temperatures, this relationship disappears, which was also confirmed in our study [47].

What is more, we have also examined the effect of hyperthermia on the efficacy cisplatin, tamoxifen, and their combination on ovarian cancer cells. It was found that the simultaneous use of cisplatin with hyperthermia has a positive impact on the drug’s cytotoxic effect on cancer cells. Treating SKOV-3 cells with the described drug and temperatures of 39 °C and 40 °C led to a decrease in their viability by 15.7% and 84.4%, respectively, compared to cells treated with cisplatin at 37 °C. These findings suggest that mild hyperthermia may enhance the efficacy of cisplatin in treating ovarian cancer.

The obtained results are consistent with the results of other studies assessing the effect of hyperthermia on the efficacy of cisplatin. In 2019, Sukovas et al. showed that the simultaneous treatment of ovarian cancer cells of the OVCAR-3 line with cisplatin and an elevated temperature led to a decrease in their viability compared to cells treated with cisplatin at a temperature of 37 °C [48]. The authors of that study also checked the effect of mild hyperthermia itself on OVCAR-3 cells. In that case, a significant reduction in cell viability was also observed [48].

In another study examining the effect of mild hyperthermia generated by gold nanorods irradiated with near red radiation (NIR) on SKOV-3 cells, it was shown that treating cancer cells with hyperthermia caused a decrease in their viability compared to the untreated cells [49]. The same study also examined the effect of mild hyperthermia on the efficacy of cisplatin cytotoxicity. It was shown that the simultaneous use of this drug in combination with hyperthermia caused a decrease in cancer cell viability by 70% compared to untreated cells [49].

The second drug whose efficacy in combination with hyperthermia on ovarian cancer cells was tested in the following study was tamoxifen. It was shown that a treatment with tamoxifen combined with a temperature of 39 °C led to a small (by approx. 1.1%) increase in cancer cell viability compared to cells treated with tamoxifen without hyperthermia. The increase was not statistically significant. The great decrease in the viability of SKOV-3 cells was observed after treatment with tamoxifen and a temperature of 40 °C—the decrease in these conditions was 83.9%, compared to cells treated with tamoxifen at a temperature of 37 °C. The obtained results indicate a positive effect of the temperature of 40 °C on the efficacy of tamoxifen in the treatment of ovarian cancer cells.

The increased cytotoxic effect of tamoxifen on cancer cells under hyperthermic conditions was confirmed in studies conducted by Piantelli et al. [50]. Their results showed that the treatment of melanoma cells with tamoxifen and elevated temperature led to a small increase in the number of apoptotic cells compared to the use of tamoxifen at a standard temperature [50].

In the following study, we also examined the effect of hyperthermia on both of the above-described drugs when used simultaneously. The combined use of cisplatin and tamoxifen with a temperature of 39 °C led to a 9% decrease in cell viability compared to these drugs used at 37 °C. The greatest decrease in cell viability was observed for the combination of cisplatin and tamoxifen and a temperature of 40 °C; a 69.6% decrease was observed when compared to cells treated with drugs at 37 °C.

The effect of combined therapy with tamoxifen and cisplatin together with hyperthermia on the development of melanoma in mice in vivo was examined in the study by Krpan et al. [51]. The use of hyperthermic temperature led to a delay in tumor growth time by about 2 days compared to the control [51]. The use of tamoxifen together with hyperthermia led to a delay in tumor growth by about 2.5 days, while with the use of cisplatin with hyperthermia it was about 7 days. The best effect of the therapy was observed while treating mice with a combination of cisplatin, tamoxifen, and hyperthermia—the delay in tumor growth time under such conditions was about 10 days [51].

The results obtained in this part of the experiment indicate that the use of combination therapy based on commonly used drugs together with hyperthermia has a positive effect on the cytotoxic activity of those drugs on ovarian cancer cells. These results are reflected in the analyzed literature.

The effect of moderate hyperthermia on ovarian cancer cells as well as on the efficacy of the cytotoxic drugs was additionally checked by assessing the morphological changes occurring in cells treated with those agents.

The treatment of SKOV-3 cells with an elevated temperature of 39 °C only resulted in an increase in the average percentage of apoptotic and necrotic cells by 7.75% compared to the control cells (cells cultured in 37 °C), while treatment with a temperature of 40 °C led to an even further increase in the percentage of apoptotic cells; this was 9.44% compared to the control. These results indicate that mild hyperthermia, used in monotherapy, has a moderate cytotoxic effect on the viability of ovarian cancer cells.

The effect of combination therapy on SKOV-3 cells was tested by the simultaneous treatment with cisplatin and elevated temperature. The use of cisplatin and a temperature of 39 °C caused an increase in the average percentage of apoptotic and necrotic cells by 9.52% compared to cells treated with cisplatin at 37 °C, while the use of this drug at 40 °C led to an increase in the number of apoptotic cells by as much as 22.51%.

In the same way, the anticancer activity of tamoxifen in combination with hyperthermia was tested. The treatment of SKOV-3 cells with the mentioned drug led to an interesting phenomenon of strong cell nuclei fragmentation, which was observed both when using a temperature of 37 °C and moderate hyperthermia. Similar changes in cell morphology following tamoxifen treatment were already observed before in HeLa cancer cell lines and included significant cell shrinkage, the condensation of cell nucleus, multinucleation, the formation of apoptotic bodies, and sporadic enucleation [52].

Cells in which this phenomenon was observed were classified as apoptotic cells, but to determine the exact cause of the nucleus fragmentation, further research is required.

The obtained results indicate that the use of tamoxifen at the standard temperature of 37 °C led to an increase in the percentage of apoptotic and necrotic cells by 85.47% compared to untreated cells. The use of tamoxifen in combination with hyperthermia resulted in a slight increase in this percentage compared to the drug used at the standard temperature, by approx. 1.5% for a temperature of 39 °C and by 2.31% for a temperature of 40 °C.

In our study, we also checked the effect of both tested drugs and moderate hyperthermia on changes in the morphology of the tested cells. The use of both drugs together with a temperature of 39 °C led to an increase in the average percentage of cells with apoptotic and necrotic features by 2.75% compared to cells treated with drugs at a temperature of 37 °C, while their use at a temperature of 40 °C resulted in a further increase by as much as 16.01%.

Interestingly, in cells treated simultaneously with cisplatin and tamoxifen, a nucleus fragmentation was not observed to such an extent as in cells treated solely with tamoxifen, which only confirms the need for further research in this area.

The results obtained in this part of the study confirm the positive effect of moderate hyperthermia on the ability of the tested drugs to induce apoptosis and necrosis in SKOV-3 ovarian cancer cells.

Due to the ability of all factors used in this study to induce autophagy processes in cancer cells, a short assessment of the induction of this phenomenon was also performed using acridine orange as a ratio metric biosensor of autophagosomes.

Autophagy is a process that enables cells to maintain homeostasis by degrading and removing damaged or unnecessary proteins or organelles in a way that allows the recovery of nutrients from them [53,54]. In the case of cancer cells, autophagy should be viewed as a process with at least a two-faceted impact on the course of the disease. In the early stages of cancer development, autophagy is a process that inhibits disease progression [53,55]. It has been shown that by removing misfolded proteins and damaged organelles, autophagy protects cells from abnormal functioning, inflammation, or abnormal antigen presentation, which predisposes cells to malignancy [53,55]. Moreover, autophagy may also contribute to the inhibition of carcinogenesis by removing certain proteins from cells, high levels of which promote cancer development (e.g., p62 protein) [53].

On the other hand, at an advanced stage of the disease, autophagy is a process that enables the survival of cancer cells and further disease development [53]. Most importantly, autophagy processes allow cancer cells to meet their metabolic needs by providing the appropriate amount of nutrients that are necessary for the further development of the tumor [56]. Autophagy also plays an important role in cancer progression, e.g., by degrading MHC-1 antigens (Major Histocompatibility Complex Class I) presented on the surface of cancer cells and thus enabling them to avoid detection by the patient’s immune system [57], by maintaining the appropriate level of ROS (Reactive Oxygen Species) in these cells [58], or by activating pathways that are key to the processes of cancer cells invasion and migration [59]. It is also worth remembering about the well-documented link between autophagy and the multidrug resistance of cancer cells. This relationship can also be considered as two-faceted, where disorders in autophagy regulations can either increase or decrease the drug resistance of malignant-transformed cells [60].

Since the activation of autophagy is likely to be an unfavorable phenomenon in carcinogenesis, the detection of autophagy induction seems to be a reasonable approach.

Our results indicate an increased induction of the autophagy process in SKOV-3 cells treated with all the tested drugs under the influence of both temperatures used in the study. The increase in autophagy induction was particularly noticeable for cells treated with drugs and a temperature of 40 °C, which was much less in the case of cells treated with drugs and a temperature of 39 °C. The most intense induction of autophagy was observed after treating cells with cisplatin at both the standard temperature of 37 °C and at both elevated temperatures.

Additionally, the effect of moderate hyperthermia alone on the autophagy process in the tested cells was also checked. It was shown that treating cells with a temperature of 39 °C led to a slight decrease in this parameter, while treatment with a temperature of 40 °C led to a significant increase.

The obtained results suggest that the temperature of 40 °C contributes to a stimulation of autophagy processes in SKOV-3 cells. It is possible that proteins from the HSP family are involved in this process, as it has been shown that these proteins (whose expression increases under heat shock conditions) are involved in the chaperone-dependent autophagy which supports the survival and progression of cancer cells in unfavorable environmental conditions, but this topic requires further research [61].

To check what mechanisms of apoptosis are affected by moderate hyperthermia in SKOV-3 cells, and to compare them with the mechanisms demonstrated by the tested drugs, an analysis of the expression of genes involved in apoptosis processes was performed using RT-qPCR. As the obtained results show, for most genes, mild hyperthermia affects their expression in the same way as the tested drugs, but to a slightly weaker extent. The most frequently observed mechanism of proapoptotic action of mild hyperthermia was inhibition in the expression of anti-apoptotic genes, including such important genes as AKT1, BCL10, BIRC2, or XIAP. This action is identical to the one shown by tamoxifen—the inhibition of the PI3K/Akt signaling pathway which activates the mTOR pathway, responsible for the inhibition of apoptosis and autophagy processes [62,63], or the degradation of the anti-apoptotic XIAP protein [64]—as well as the one shown by cisplatin—the inhibition of the expression of BNIP2 [65] and XIAP [66] genes. These results allow us to assume that the simultaneous use of both hyperthermia and drugs will lead to an even stronger proapoptotic effect on cancer cells than the use of these agents in monotherapy.

Additionally, the inhibitory effect of mild hyperthermia on the expression of some of the proapoptotic genes may also contribute to reducing the resistance of cancer cells to cytotoxic drugs, because the overexpression of these genes is often associated with the occurrence of drug resistance in cancer cells. A good example of such a correlation is the XIAP gene—one of the most important regulators of cisplatin-induced apoptosis [67]. Ovarian cancer cells often overexpress this gene, which in turn leads to their resistance to cisplatin treatment. It has been proven that inhibiting or completely silencing the expression of the XIAP gene leads to an increased sensitivity of cells to cisplatin and promotes apoptosis in cancer cells [67].

Meanwhile, in cisplatin-resistant gastric cancer cells, the activity of another anti-apoptotic gene—AKT1, an element of the PI3K/Akt signaling pathway (involved in the regulation of cell proliferation, death, and survival)—increases in response to cisplatin treatment [68]. The increased activity of this gene leads to the higher activity of the aforementioned signaling pathway, which in consequence results in a decrease in the cytotoxic effect of cisplatin. The inhibition of the AKT1 gene leads to an increased chemosensitivity of cancer cells to cisplatin, and thus to the increase in its effectiveness [68]. As mentioned earlier in this manuscript, tamoxifen, by inducing the expression of proapoptotic genes, also has the ability to regulate the PI3K/Akt/MAPK signaling pathway, which suggests that its combined use with hyperthermia may additionally enhance its cytotoxic effect on cancer cells [11].

Additionally, as our results show, moderate hyperthermia can also lead to an increase in the expression of some proapoptotic genes, including genes encoding caspases, e.g., CASP3, CASP9, as well as FAS, HRK, and RIPK2 genes. This effect was not observed in cells treated with the tested drugs.

Also, for this group of genes, an increase in their expression induced by hyperthermia may lead to a decrease in the resistance of cancer cells to cytotoxic drugs. An example of this mechanism is the expression of the FAS gene in lung cancer cells. The FAS gene encodes the death receptor, which participates in the extrinsic apoptosis pathway [69]. The increased expression of this gene leads to the down-regulation of genes involved in cisplatin resistance—GSTP1, involved in the mechanism of intracellular inactivation of cisplatin, and the ERCC1 gene, which encodes a protein involved in the repair of DNA damage induced by cisplatin. The reduced activity of the mentioned genes results in an increased sensitivity of cancer cells to cisplatin [69]. Another proapoptotic gene, the increased expression of which contributes to the effectiveness of cisplatin is the CASP3 gene, which encodes caspase 3 [70]. Caspase 3 is the main apoptosis execution enzyme that mediates the process of apoptosis induced by cisplatin. It has been shown that the increased expression of the CASP3 gene leads to an increased cytotoxic effect of cisplatin on cancer cells resistant to this drug [70].

The provided examples indicate that although the use of hyperthermia against cancer cells does not lead to such spectacular effects as those observed after the use of cytotoxic drugs, combining this agent with drugs may contribute to a significant improvement in their effectiveness against cancer cells.

## 5. Summary

The results obtained in the above study suggest that mild hyperthermia enhances the cytotoxic effect of cisplatin, tamoxifen, and a combination of both drugs on SKOV-3 ovarian cancer cells. The elevated temperatures of 39 °C and 40 °C applied independently also demonstrate toxic effects on these cells, but with a much lower intensity than the above-mentioned drugs.

The main molecular mechanisms used to activate apoptosis processes in SKOV-3 cells by drugs and temperature are the inhibition of anti-apoptotic genes, and in the case of moderate hyperthermia, the induction of the expression of proapoptotic genes. Nevertheless, the potential limitation of hyperthermia in ovarian cancer therapy may be the activation of autophagy processes observed in cells during treatment with elevated temperatures. However, it seems that this obstacle may be controllable. Due to limited knowledge in terms of the possible practical application of the described therapy, further research in this area is required, especially in the aspect of details regarding the induction of the autophagy process.

## Data Availability

The datasets used and/or analyzed during the current study are available from the corresponding author on reasonable request.

## Supplementary Materials

The following supporting information can be downloaded at: www.mdpi.com/article/10.3390/ijms252413664/s1.
