# Supplementary material for: Hyperthermia Potentiates the Effectiveness of Anticancer Drugs—Cisplatin and Tamoxifen on Ovarian Cancer Cells In Vitro
Source: Int J Mol Sci. 2024 Dec 20;25(24):13664. doi: 10.3390/ijms252413664 (PMC11728268; doi:10.3390/ijms252413664)
Supplement: Supplementary file 1 [file ijms-25-13664-s001.zip › ijms-3369169-supplementary.pdf]

Table S1. Average percentage viability of SKOV-3 cells treated with mild hyperthermia for 24 h and drugs, obtained in MTT assay.

	**Temperature**	**Cisplatin**	**Tamoxifen**	**Cisplatin + Tamoxifen**
**37°C**	100% (control)	84,82+/- 0,11%	86,46+/-0,21%	70,24+/-0.08%
**39°C**	107,1+/-0,04%	69,14+/-0,05%	87,63+/-0,08%	61,18+/-0,03%
**40°C**	5,54+/-0,04%	0,44+/-0,01%	2,61+/-0,02%	0,59+/-0,01%

Table S2. Summary of the average percentage of apoptotic and necrotic SKOV-3 cells treated with mild hyperthermia and drugs, stained with acridine orange.

	**Temperature**	**Cisplatin**	**Tamoxifen**	**Cisplatin + Tamoxifen**
**37°C**	8,46±2,46	16,57±3,51	93,93±1,64	30,10±7,59
**39°C**	16,21±3,30	26,09±2,85	95,40±1,63	32,85±2,54
**40°C**	17,90±4,03	39,08±4,06	96,24±0,85	46,11±4,36
